# Conceptions of geography and history as school disciplines: an approach from lexical availability

**DOI:** 10.3389/fpsyg.2023.1263421

**Published:** 2023-08-31

**Authors:** Juan Luis de la Montaña Conchiña, Guadalupe de la Maya Retamar, Magdalena López-Pérez

**Affiliations:** Faculty of Education and Psychology, Department of Didactics of Social Sciences, Languages and Literatures, University of Extremadura, Badajoz, Spain

**Keywords:** lexical availability, social sciences, history, geography, conceptual evolution, bilingual education

## Abstract

This paper aims to examine students’ conceptions of Geography and History as school disciplines at different educational stages. The sample, composed by a total of 73 participants from Primary School (*n* = 26), Secondary School (*n* = 29) and Higher Education (*n* = 18), completed a lexical availability test in Spanish and English or in Spanish and French. The results show that lexical availability increases as the educational stage increases, although the differences are not significant between all of them. The available lexical items on Geography and History, most of which are not shared between the different stages, are very generalist, showing a rigid and formal view of the disciplines. After the analysis carried out, we consider that lexical availability may constitute a valid tool for accessing students’ conceptions.

## 1. Introduction

The initial development of lexical availability is framed in the work for the elaboration of the *Français Fondammental* (Gougenheim et al., 1956), a basic vocabulary intended to facilitate the acquisition of French by foreigners, when the lexical selection criterion of frequency was joined to that of available vocabulary, defined by López-Morales (1995–1996, p. 245) as “the set of words that speakers have in their mental lexicon and whose use is conditioned by the specific subject of the communication.” Thus, from associative tests carried out around certain prompts, a related vocabulary emerges: “the potential lexicon that belongs to the active stock of the subjects” (Ávila and Sánchez, 2011, p. 47) and which is used when the need arises. Since that early work, studies have diversified both theoretically and methodologically. On the theoretical level, the approaches have been gaining in depth and applicability to varied fields such as dialectology, linguistics, psycholinguistics, ethnolinguistics and the teaching of both native and foreign languages. With regard to methodological developments, the work carried out in the Spanish-speaking world, initially promoted by López-Morales (1995–1996), has contributed not only to the establishment of common guidelines in terms of the samples, the prompts surveyed or the development of the tests, but also to an improvement in the statistical treatment of the data and to the development of mathematical formulae to adequately explain the relationship between knowledge and lexical production.

If, as we explained above, the applications of lexical availability are diverse, in the field of education, its contributions have shown the influence of certain variables on the lexical availability, the differences in this between students and teachers or its usefulness for assessment and teaching (Zambrano et al., 2019). Likewise, as Santos and Juárez (2022) point out, the methodological bases of availability have been used to study students’ perception of reality (Ávila et al., 2020) as well as their conceptions of education (Santos and Juárez, 2022) or reading and literary education (Juárez, 2019; Trigo and Santos, 2023).

Students’ ideas or conceptions are a particularly relevant issue for didactics because, as García (2002) points out, they directly affect the consideration of school knowledge, both in its design and construction process. In this way, this study strengthens the line of lexical availability that explores the conceptions of students (high school and university) regarding topics of specific disciplines, such as Geography, History, Education, Reading, Mathematics, Algebra, etc. (*cf.* Ferreira et al., 2014; Pacheco et al., 2016; Martínez-Lara, 2021). More specifically, knowledge of conceptions about scientific content or about certain school disciplines, in our case the Social Sciences (Geography and History), is a fundamental aspect that can be investigated from the initial stages of the schooling process to the end of higher education (Feixas, 2010). In this respect, the didactic research carried out has been decisive in understanding students’ and teachers’ conceptions of the disciplines with which they will work and learn, given that these can be determining factors both in their learning and in classroom practice. Let us not forget that when we talk about conceptions we are talking about implicit social, cultural, environmental, and academic elements that interrelate and materialize in the different actions of individuals (Machuca, 2012).

Geography and History are core subjects of exceptional formative power present from the initial educational cycles. Research has resulted in an abundance of scientific literature in which conceptions of this subject do not focus exclusively on how it is understood by students or future teachers. Thus, in the field of students, transversal and longitudinal research has been carried out on disciplinary conceptions and the change or evolution of these conceptions (Fuentes, 2004; Vera and Cubillos, 2010; Fraile-Delgado, 2020), as these conceptions are mainly mediated by video games, social networks, and cinema. Similarly, and in the field of teacher training, research has been carried out on conceptions referring to disciplinary epistemology*, about* and *of* History (Suárez, 2012; Montaña, 2016; Gómez et al., 2018; Martínez-Hita and Miralles, 2021); also on content specific to each subject –controversial topics, heritage, gender, etc.– (Cuenca and Estepa, 2007; López-Zurita and Felices, 2023) or educational assessment, resources and procedures and textbooks (López-Sánchez et al., 2018). We cannot forget that, in this context of initial training, knowing the conceptions allows, in the first instance, to visualize, intervene and modify them, letting the trainee teacher change the vision of the discipline and his or her work in the classroom (Ibagón-Martín, 2020).

Access to conceptions has traditionally been carried out following qualitative approaches, through questionnaires with open-ended questions that seek to get respondents to make explicit their ideas about the disciplines. However, in this paper we propose an approach to these conceptions through lexical availability, understood as the tool or technique that allows us to obtain the available lexicon (Hernández and Tomé, 2017), with the aim of examining the conceptions that students from different educational stages have about Social Sciences –Geography and History– as a school discipline, trying to categorize their available lexicon in this regard and analysing the evolution through the different educational stages.

In this paper we present the preliminary results of a study carried out with students following a bilingual education in French or English, in which Geography and History are partially taught in these languages. More specifically, we have analysed the lexical availability not only about both disciplines, but also about some prompts that are related to the thematic areas that structure them, although, at this point, we will focus on the results from Spanish and only about the *Geography and History* prompts.

## 2. Methods

The research design used in this piece of research is cross-sectional, descriptive, and non-correlational. The methodology of analysis is mixed, presenting both quantitative and qualitative results. The sampling carried out was not probabilistic, as the students were chosen intentionally, since, for the purposes of the research, data were collected from students who were taking Social Studies subjects in a foreign language, in the context of the bilingual programmes implemented in the region of Extremadura.

### 2.1. Sample

The sample of the present research was composed by 73 students (*n* = 73), who are taking a subject related to Social Sciences: *Social Sciences*, in the 6th year of Primary Education (*n* = 26), belonging to a bilingual section Spanish-French (group 1); *Geography and History* in the 3rd year of Compulsory Secondary Education (*n* = 29), belonging to a bilingual section Spanish-English (group 2), and *Didactics of Social Sciences*, in the 2nd year of the University Primary Education Degree (*n* = 18), also belonging to a bilingual training Spanish-English (group 3). As a whole, the sample shows a balance between the genders, although the percentage of male participants is somewhat higher (55.3%). The mean ages were 11.5 years old in group 1, 14.5 in group 2 and 21.6 in group 3. Since the sample is not representative of the universe explored, the results just serve as a first approximation, but they cannot be generalized.

### 2.2. Instrument

The data obtained in the present study come from two instruments: a sociological questionnaire with questions relating to gender, age, academic year, mother tongue, languages in which they take their subjects and activities carried out in those languages, and a lexical availability test. This is an associative test in which, starting from a stimulus, students have to come up with the lexical item that comes to their mind, associated with that stimulus. The test, as set out in the *Pan-Hispanic Lexical Availability Project*, is based on the open list system, and allows 2 min to complete the lexicon for each prompt (López-González, 2014). The students were introduced six prompts: *Geography*, *Physical environment*, *Population*, *History*, *Prehistory* and *Middle Ages*, which they completed firstly in Spanish and later in English/French, although, in this paper, it is only dealt with the Spanish results of prompts 1 (*Geography*) and 4 (*History*).

### 2.3. Procedure and data analysis

The tests were administered in the second and third trimester of the 2022–23 academic year, in a single session. After collecting informed consent for participation in the study, which was signed by parents in the case of minors, participants completed the sociological questionnaire and then the availability test. In this, the prompts were presented orally and in writing and they were given 2 min to complete each of them, first in Spanish and then in a foreign language. They were instructed not to worry about spelling correction to avoid this affecting lexical productivity.

Once the lexicon was retrieved, it was edited according to the guidelines established by Samper (1998) for Spanish. It was also decided to admit all the words evoked by informants, for better understanding of the relationships between words, in accordance with the criteria retained by Trigo and Santos (2023). The data were processed with the *Dispogen II* package (Echeverría et al., 2005) for the specific calculations of availability; with *IBM SPSS Statistics* v. 23, for the statistical analysis taking into consideration the variables analysed, and with *Atlas.ti* for the qualitative analysis and the generation of word clouds based on frequency.

## 3. Results

A total of 2009 words were updated by the participants, of which 1,056 belonged to prompt 1 for *Geography* and 953 to prompt 4 for *History*. The total number of tokens was 736, with more words in the first prompt (400) than in the second one (336). Table 1 shows the data specified according to the stage of education the students are at.

**Table 1:** Number of tokens and types by educational stages.

		Geography				History			
	*N*	Tokens	Types	Mean	Cohesion index	Tokens	Types	Mean	Cohesion index
Primary	26	314	143	12.07	0.08	261	112	10.03	0.08
Secondary	29	448	202	15.44	0.07	415	250	14.31	0.05
Higher education	18	294	124	16.33	0.13	277	164	15.38	0.09
Total	73	1,056	336	14.46	0.04	953	400	13.05	0.03

As it can be seen, the average production in both prompts increases from one educational stage to the next, although the differences are more marked in the transition from Primary to Secondary, between which there are three academic years, than between the latter and Higher Education, between which there are 4 years of difference. Likewise, although the trend is identical between the two prompts, it is in *Geography* where the values are higher, both in tokens and in the average number of answers per respondent. In order to determine whether these differences by educational stage are statistically significant, the assumptions that must be met by the data series were verified, and accepted in the case of *Geography*, the one-factor ANOVA test was carried out, while in *History*, as the randomization assumption was not met, the non-parametric Kruskal-Wallis test was performed. The results show that, in the *Geography* prompt, there are differences in the total word production taking into account the educational stage in which they are (*F* = 3.235; *p* = 0.045). The post-hoc analyses, using the Scheffé test, do not reveal the existence of differences between groups, probably because the *p* value is at the limit of significance. T-tests for group comparisons using Student’s *T* test do reveal the existence of differences between students in Primary and Secondary Education (*t* = −2.013; *p* = 0.049) and those in Primary and Higher Education (*t* = −2.290; *p* = 0.027). On the other hand, in the centre of *History* there are also differences between groups (*H* = 14.667; *p* = 0.001), specifically between Primary and Secondary Education (*Z* = -3.061; *p* = 0.002) and Primary and Higher Education (*Z* = -3.435; *p* = 0.001), but not between the productive vocabulary, measured through lexical availability, of Secondary and Higher Education students (*Z* = -0.791; *p* = 0.429).

In terms of lexical density, the result of dividing the total number of tokens by the number of types, it is also higher in Geography than in History, with the sole exception of the data for Primary School, where the density in History is slightly higher than in Geography. In this prompt, the data are more homogeneous as, overall and in each of the groups, the cohesion index is higher.

For the qualitative analysis, it has been taken into consideration the 10 most available words in the three groups, which are listed in Table 2.

**Table 2:** Most available words by educational stage.

Geography			History		
Primary E.	Secondary E.	Higher E.	Primary E.	Secondary E.	Higher E.
Earth	Country	Mountain	Middle ages	War	King
Planet	Map	Relief	The discovery of America	King	War
City	Continent	Map	Ancient history	Napoleon	Conquest
Spain	Mountain	River	Modern age	French revolution	Queen
Continent	River	Climate	Prehistory	Prehistory	Castle
Mountain	City	Fauna	Metal ages	Past	Evolution
City	Spain	Country	Contemporary age	Ancient	Discovery
Sun	Sea	Lake	War	Palaeolithic	Neolithic
Solar system	World	Flora	Neolithic	Rome	Palaeolithic
Rome	Lake	Hydrography	King	Time	Stage

Focusing on the *Geography* prompt, it is found that, among these most available words, only two words are present at all stages: country and mountain. Primary and Secondary students also share three words (Spain, continent, and city), the same as Secondary and Higher Education students (lake, map, and river), but students at the two most distant stages have no words in common other than those listed as shared by all.

In the History prompt, on the other hand, only two words common to the three educational stages have been found: king and war, which, although they appear in the last positions in Primary, are in the first two positions in the upper stages. In this prompt, in addition to these two words, the different stages share only one word: neolithic (Primary-Higher Education), prehistory (Primary-Secondary Education) and palaeolithic (Secondary-Higher Education).

**Figure 1:**
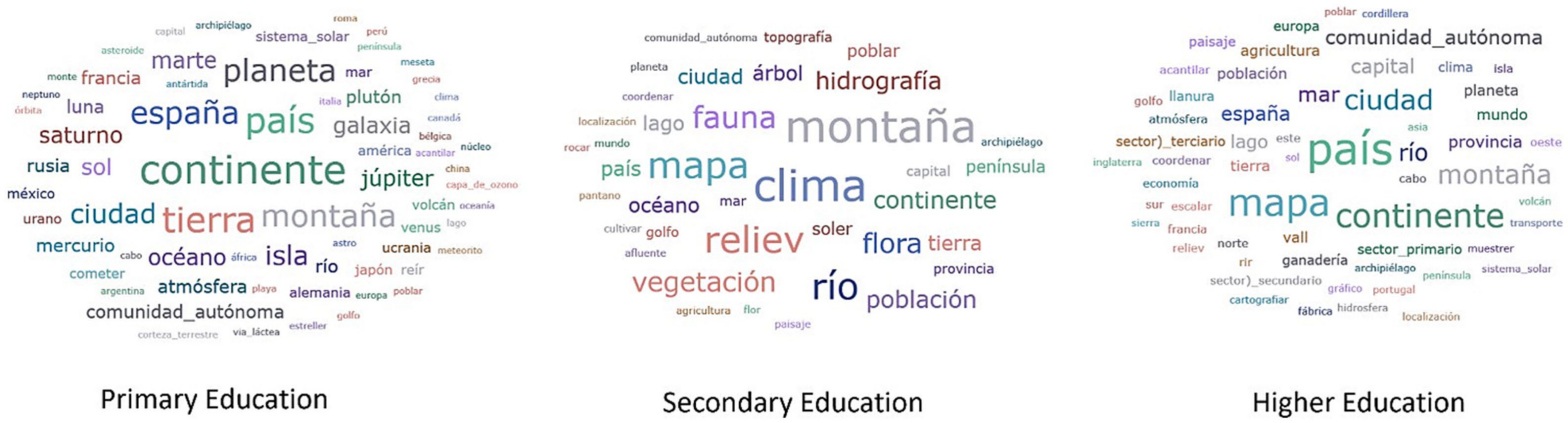
Geography lexicon according to frequency.

**Figure 2:**
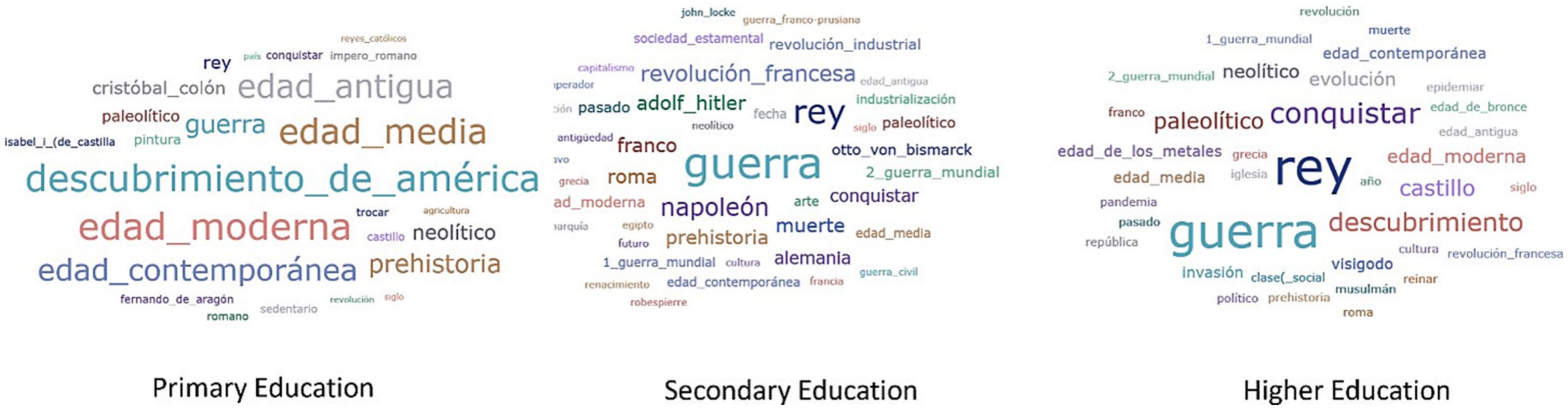
History lexicon according to frequency.

In relation to frequency, Figures 1, 2 present word clouds in which the larger ones indicate their more frequent character.

In Geography, the predominant lexicon is that derived from physical geography of a descriptive nature, an aspect confirmed by the predominance of terms such as mountain, continent, atmosphere, ocean, hydrography, vegetation. Terms related to political geography (borders, limits, administrative units) such as country, Spain, Autonomous Community, province, capital city also stand out.

In History, the terms oscillate in three areas: those related to the classical temporal organization of the different stages of history, names of important historical figures and historical events. The division of historical periods –Prehistory, Ancient, Middle Ages, Modern and Contemporary– seems to be related to the citation of certain characters, such as Napoleon, Hitler, Franco or Columbus, as well as the exclusively human phenomenon of war. No reference to women is found. Historical events include the French Revolution, the Industrial Revolution and, especially, the Discovery of America.

## 4. Discussion and conclusions

From the analysis of the above data, it can be seen that the available lexis is somewhat higher in *Geography* than in *History*, and that both increase as the educational stage of study increases. Although there are no previous studies that have been interested in lexis related to school disciplines, in general, with reference to the lexis of the prompts surveyed by the traditional studies of lexical availability, the increase in lexis as the level of education rises is a constant in both mother tongue and foreign languages (Carcedo, 2000; Galloso and Prado, 2012; Herranz-Llácer and Marcos-Calvo, 2022). It is also logical to think that, as the block of contents on subjects related to the social sciences are addressed, the lexis studied will be greater, since the contents are diversified and studied in greater depth. However, although the differences between the Primary and Secondary stages are significant, it is striking that the lexis available increases from one stage to the next by only three or four words (Escudero et al., 2022).

Another interesting issue is the lack of shared lexicon among students at different stages. Given that the lexis available seems to be permeable to external factors (Galloso and Prado, 2012), it is normal that the lexis updated by the respondents responds to the content being worked on at the time, as shown by the fact, for example, in History, of including events or historical figures already cited which coincide with the basic knowledge of the teaching programme for that year. But, to a certain extent, it seems that with each new year of study, pupils make a clean sweep of what they have previously internalized and do not evoke in their available lexicon certain words that could be considered key.

As far as the students’ conceptions of the disciplines are concerned, it must be pointed out that the differences are minimal with respect to other studies focusing exclusively on the conceptual sphere. The use of an extraordinarily generalist lexicon suggests that pupils have a rigid and excessively formal view of the disciplines from the most elementary stages of education. Thus, in the case of Geography, the use of terms such as continent, Spain or country place us before a merely descriptive Geography, typical of basic educational cycles, far removed from the current problems set out in the SDGs (García, 2002). However, the similarity of the lexicon collected in the university context suggests a continuity in the conceptual field that has not been substantially modified in the intermediate cycle (Secondary), despite registering a significant and improved increase in vocabulary.

Similar results can be found in the field of History. The words collected convey a linear vision of history in which the synchronic ordering of the stages of the past occupies a relevant place, at least in the initial cycles (Ancient, Medieval, Modern, Contemporary). In the same way, terms such as war, king, conquest, the mention of certain characters linked to specific events such as the First and Second World Wars and, especially, the Discovery of America (1492) occupy a relevant place. This allows us to affirm that factual, descriptive (López-Zurita and Felices, 2023), teleological and possibly uncritical elements underlie the reductionist views of the discipline that are not only maintained in the different educational cycles (Gómez et al., 2018; Ibagón-Martín, 2020), but that probably resist possible attempts at modification developed didactically in university contexts (Montaña, 2016).

Since this work is a preliminary presentation of results, it will be necessary in future work to go into them in greater depth, introducing, in addition, a comparison between the lexis available in Spanish and in the foreign language in which the students study these disciplines when they participate in bilingual teaching programmes. Also, it will be necessary to continue exploring this line with a broader population to verify whether the results of this research mark a trend in the autonomous community of Extremadura.

## Data availability statement

The raw data supporting the conclusions of this article will be made available by the authors, without undue reservation.

## Ethics statement

The studies involving humans were approved by the Bioethics and Biosecurity Committee of the University of Extremadura (No. 30/2023). The studies were conducted in accordance with the local legislation and institutional requirements. Written informed consent for participation in this study was provided by the participants themselves or their legal guardians/next of kin.

## Author contributions

JM, GM, and ML contributed to conception and design of the study. ML organized the database. GM performed the statistical analysis. JM wrote the first draft of the manuscript. JM, GM, and ML wrote sections of the manuscript. All authors contributed to manuscript revision, read, and approved the submitted version.

## Funding

The author(s) declare financial support was received for the research, authorship, and/or publication of this article.

This research was funded by the Regional Ministry of Economy, Science and the Digital Agenda of the Regional Government of Extremadura (Consejería de Economía, Ciencia y Agenda Digital de la Junta de Extremadura) and by the European Union. Project number IB20074.

## Conflict of interest

The authors declare that the research was conducted in the absence of any commercial or financial relationships that could be construed as a potential conflict of interest.

## Publisher’s note

All claims expressed in this article are solely those of the authors and do not necessarily represent those of their affiliated organizations, or those of the publisher, the editors and the reviewers. Any product that may be evaluated in this article, or claim that may be made by its manufacturer, is not guaranteed or endorsed by the publisher.
